# Microbiome-Metabolome Responses to a High-Grain Diet Associated with the Hind-Gut Health of Goats

**DOI:** 10.3389/fmicb.2017.01764

**Published:** 2017-09-14

**Authors:** Shiyu Tao, Ping Tian, Yanwen Luo, Jing Tian, Canfeng Hua, Yali Geng, Rihua Cong, Yingdong Ni, Ruqian Zhao

**Affiliations:** ^1^Key Laboratory of Animal Physiology and Biochemistry, Ministry of Agriculture, Nanjing Agricultural University Nanjing, China; ^2^Animal physiology teaching and research office, College of Veterinary Medicine, Northwest A & F University Yangling, China

**Keywords:** microbiome, metabolome, high-concentrate diet, host health, hindgut, goats

## Abstract

Studies on the effect of a high-concentrate (HC) diet on the hindgut microbiota and metabolome of ruminants are rarely reported. We used 454 pyrosequencing of 16S rDNA genes and gas chromatography-mass spectrometry to evaluate the effects of long-term feeding (HL) or short-term (HS) feeding of an HC diet on changes in bacterial microbiota and their metabolites in the hindgut, with Guanzhong goat as a ruminant model. Results indicated that an HC diet decreased bacterial diversity and induced metabolic disorder in the hindgut. The levels of lactate, endotoxin (lipopolysaccharide, LPS), and volatile fatty acid concentrations were higher in the intestinal digesta of the HC goats than in those of the LC goats (*P* < 0.05). The level of beta-alanine decreased, whereas the levels of stigmasterol and quinic acid decreased in the cecal and colonic digesta of the HC goats. At the genus level, the abundance of *Clostridium* and *Turicibacter* was significantly increased in both the colonic and cecal digesta of the HC goats. Several potential relationships between metabolites and several microbial species were revealed in this study. The mRNA expression of the genes functionally associated with nutrients transport, including *NHE2, NHE3, MCT1*, and *MCT4* were significantly downregulated in the colonic mucosa by the HC diet (*P* < 0.05). The expression levels of the genes related to the inflammatory response, including *TLR4, MYD88, TNF-*α, and *IL-1*β were markedly upregulated in the cecal mucosa by the HC diet (*P* < 0.05). Our results indicate that an HC diet induces microbiota dysbiosis, metabolic disorders, and mucosal damage in the hindgut of goats.

## Introduction

To meet the required energy intake for high milk production, a common strategy is to feed a large amount of dietary grain or easily degradable dietary byproducts to lactating dairy cows. However, the excessive amounts of carbohydrates and highly fermentable forage increase the possibility of developing subacute ruminal acidosis (SARA) and decreases long-term production (Plaizier et al., 2008; Zhang et al., 2016). The effects of a high-concentrate (HC) diet on ruminal short-chain fatty acid (SCFA) accumulation, ruminal pH depression, ruminal microbiota dysbiosis, and host health have recently drawn increased interest (Dong et al., 2013; Mao et al., 2016). Processes that occur in the rumen of animals with SARA are also observed in the hindgut (colon and cecum). As ruminants, the anatomy and the physiological functions of the digestive tracts are conserved between cattle and goat. Due to the small size, low price and the convenience for sampling, dairy goats are commonly used as the animal model for investigating the mechanisms behind the metabolic disorders occur in dairy cows. In our previous studies, feeding an HC diet to lactating goats induced increased concentrations of SCFA and reduced pH-values, as well as increased lipoplysaccharide (LPS) content in the hindgut lumen (Li et al., 2012). These alterations may alter microflora composition in the hindgut, particularly for microbiota susceptible to low pH (Gressley et al., 2011; Metzler-Zebeli et al., 2013b). Short-term feeding of an HC diet to goats affected the bacterial communities in the hindgut (Liu et al., 2014; Ye et al., 2016). However, studies on the effects of a long-term HC diet on the microbial composition and epithelial functions of the hindgut have not been reported.

Some harmful metabolites in the rumen are transported toward the downstream organs, including the liver (Chang et al., 2015) and the mammary gland (Colman et al., 2010), eventually impairing the health of the host. Rumen metabolic perturbations accompanied with ruminal dysbiosis are important risk factors for developing diseases, particularly metabolic diseases, such as mastitis, abomasal displacement, and laminitis (Plaizier et al., 2008). However, compared with the rumen epithelium coated with a stratum corneum layer and multicellular layers in the middle (Graham and Simmons, 2005), the hindgut epithelium is considerably more “leaky” because of the monolayer structure (Dong et al., 2011). Accordingly, a high risk of harmful metabolites was being translocated from the hindgut epithelium to peripheral systems when ruminants were fed HC diets. As a physical barrier, the intestinal epithelial mucosa separates toxic compounds from deeper intestinal layers (Turner, 2009). Thus, to prevent microbiome-associated harmful metabolites from damaging the intestinal or distant organs, the structural and functional integrity of the mucosal epithelium needs to be maintained. Clinically, high-grain feeding induces diarrhea and frothy feces and increases the particle size of feces (Gressley et al., 2011; Li et al., 2012). Experimentally, a high-grain diet induces apoptosis in epithelial cells, decreases stem cell proliferation and disrupts tight junction proteins in the hindgut mucosa (Tao et al., 2014b, 2016). Therefore, the current study aimed to investigate the changes in fermented products and toxic compounds, microbiome, metabolomics, and mucosal status of the hindgut of lactating goats fed with an HC diet over a short or long period.

## Results

### High-concentrate diet induced changes in SCFA and LPS content in hindgut digesta

As shown in Table 1, compared with the LC group, the HS, and HL groups contained significantly higher free LPS concentrations in the colonic digesta (*P* < 0.05). Moreover, the HS goats showed increases in lactate, acetate, propionate, isobutyrate, butyrate, valerate and total SCFA in the colonic digesta relative to the LC goats (*P* < 0.05). Similarly, compared with the LC goats, the HL goats had higher concentrations of acetate, propionate, and total SCFA in the colonic digesta (*P* < 0.05). In the cecal digesta, the HS and HL goats showed higher levels of free LPS and lactate than those of the LC goats (*P* < 0.05). The concentrations of propionate, butyrate, and total SCFA in the cecal digesta were significantly increased in the HS goats than in the LC goats (*P* < 0.05). However, no significant difference in SCFA content in the cecal digesta was detected between the HL group and the LC group (*P* > 0.05).

**Table 1:** LPS, SCFA, and lactate concentration in the hindgut digesta in goats fed high concentrate diet.

**Items**	**LC**	**HS**	**HL**
**COLONIC DIGESTA**			
LPS (EU/g)	52376.15 ± 3898.82	71372.80 ± 1470.78^*^	62950.25 ± 1651.05^*^
Acetate (μmol/g)	4.70 ± 0.23	6.61 ± 0.83^*^	6.03 ± 0.23^*^
Propionate (μmol/g)	1.83 ± 0.10	3.33 ± 0.46^*^	2.46 ± 0.13^*^
Isobutyrate (μmol/g)	0.16 ± 0.01	0.20 ± 0.02^*^	0.17 ± 0.01
Butyrate (μmol/g)	1.24 ± 0.08	2.14 ± 0.02^*^	1.55 ± 0.15
Isovalerate (μmol/g)	0.14 ± 0.01	0.16 ± 0.11	0.14 ± 0.01
Valerate (μmol/g)	0.20 ± 0.01	0.29 ± 0.03^*^	0.24 ± 0.02
Total SCFA (μmol/g)	8.27 ± 0.31	12.78 ± 1.52^*^	10.62 ± 0.41^*^
Lactate (μmol/g)	0.85 ± 0.16	1.22 ± 0.07^*^	1.09 ± 0.22
**CAECAL DIGESTA**			
LPS (EU/g)	56679.26 ± 2009.05	61977.33 ± 1397.28^*^	68664.99 ± 2857.02^*^
Acetate (μmol/g)	5.12 ± 0.39	6.28 ± 0.72	5.73 ± 0.32
Propionate (μmol/g)	2.03 ± 0.21	2.89 ± 0.26^*^	2.40 ± 0.11
Isobutyrate (μmol/g)	0.17 ± 0.01	0.18 ± 0.16	0.17 ± 0.01
Butyrate (μmol/g)	1.32 ± 0.98	1.87 ± 0.20^*^	1.65 ± 0.22
Isovalerate (μmol/g)	0.15 ± 0.01	0.15 ± 0.02	0.15 ± 0.01
Valerate (μmol/g)	0.24 ± 0.01	0.27 ± 0.03	0.29 ± 0.02
Total SCFA (μmol/g)	9.09 ± 0.65	11.69 ± 1.14^*^	10.48 ± 0.66
Lactate (μmol/g)	0.35 ± 0.05	0.98 ± 0.28^*^	0.99 ± 0.18^*^

*Values are mean ± SEM, n = 5*.

* *P < 0.05 vs. LC*.

### Multivariate analysis of hindgut digesta compounds

**Figure 1:**
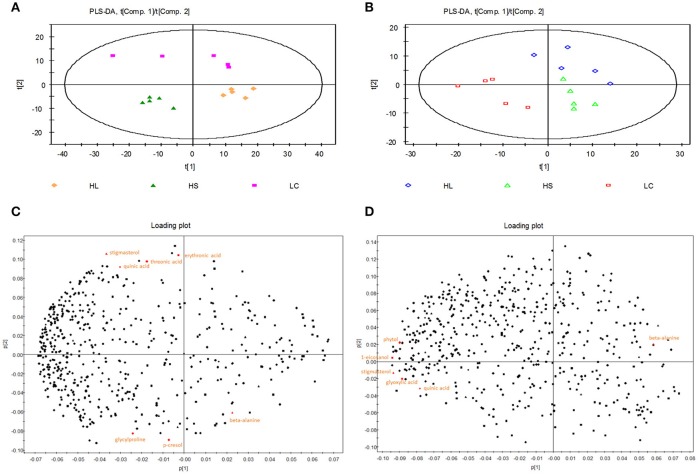
Partial least squares discriminant analysis (PLS-DA) and loading plot based on the data of the hindgut digesta compounds. **(A)** PLS-DA score plots discriminating between the colonic digesta of goats fed LC (pink square), HS (green triangle), and HL (orange rhombus) diet. **(B)** PLS-DA score plots discriminating between the caecal digesta of goats fed LC (red square), HS (green triangle), and HL (blue rhombus) diets. Loading plot of the 7 and 6 significant metabolites in the colonic **(C)** and caecal **(D)** digesta projected into the PLS-DA model compounds are labeled by the names used in Table 2.

Partial least squares-discriminant analysis (PLS-DA) was employed to identify the key compounds to which the differences among the various groups are attributed. Score plots found on the first 2 components showed that samples from the LC, HS, and HL groups were well separated (Figure 1).

The PLS-DA loading plot was generated to show the specific metabolites of the three groups, as presented in Figures 1C,D. The compounds responsible for the respective difference between the LC and HS groups or between the LC and HL groups were identified in this study. Seven compounds with variable importance in the projection (VIP) > 1.4 were identified as major distinguishing substances in the colonic digesta compounds among the 3 groups (Table 2). Three of these compounds (beta-alanine, p-cresol, and glycylproline) were markedly increased, whereas 4 (threonic acid, stigmasterol, erythronic acid, and quinic acid) were decreased by the HC diet relative to the control. Six compounds with VIP > 1.4 were recognized as the differentiating substances in the cecal digesta compounds among the 3 groups (Table 2). Only one of these compounds (beta-alanine) was increased, whereas the remaining 5 compounds (glyoxylic acid, stigmasterol, 1-eicosanol, phytol, and quinic acid) were decreased by the HC diet relative to the control.

**Table 2:** Candidate hindgut digesta compounds that differed between the control and treatment.

**Compounds**	**LC vs. HS**			**LC vs. HL**		
	**VIP**	***p*-value**	**FC**	**VIP**	***p*-value**	**FC**
**COLONIC DIGESTA**						
beta-alanine	1.46	0.016	1.09	1.84	0.032	0.95
threonic acid	1.60	0.016	−1.43	1.70	0.032	−1.37
p-cresol	1.69	0.016	2.09	1.34	0.016	2.05
stigmasterol	1.79	0.008	−1.18	1.90	0.008	−1.18
erythronic acid	1.81	0.008	−1.59	1.78	0.008	−1.63
glycylproline	1.61	0.016	0.67	1.47	0.032	0.50
quinic acid	1.56	0.008	−1.13	1.67	0.016	−0.76
**CAECAL DIGESTA**						
beta-alanine	1.43	0.008	1.03	1.79	0.008	1.10
glyoxylic acid	1.85	0.016	−0.88	1.95	0.008	−1.05
stigmasterol	2.07	0.008	−1.50	1.90	0.016	−1.01
1-eicosanol	1.88	0.008	−1.06	1.99	0.008	−1.37
phytol	1.81	0.008	−1.54	1.83	0.016	−1.11
quinic acid	2.14	0.008	−1.30	1.60	0.016	−1.07

*VIP, Variable Importance in the Projection, was obtained from the OPLS-DA model. The p-value was calculated from Student's t-test. n = 5. FC, fold change, was calculated as a binary logarithm of the average mass response (normalized peak area) ratio between Group LC vs. Group HS (HL), where a positive value means that the average mass response of the metabolite in Group HS (HL) is larger than that in Group LC and a negative value means that the average mass response of the metabolite in Group LC is larger than that in Group HS (HL)*.

### High concentrate diet induced microbiome changes in the hindgut digesta

In total, 863,237 and 876,900 reads were obtained for the bacterial 16S rRNA genes by pyrosequencing analysis in the colonic and cecal digesta, respectively. After screening these gene sequences in accordance with a strict set of criteria, 676,115 and 690,931 high-quality reads were obtained comprising for 78.32 and 78.80% of the raw reads in the colonic and cecal digesta, respectively. As shown in Table 3, the ACE, Chao 1, and Shannon indices were significantly decreased in the hindgut digesta of the HL group relative to those of the LC group (*P* < 0.05). Compared with the LC group, the HS goats showed a lower Shannon index in the colonic digesta, as well as ACE and Chao 1 indices in the cecal digesta (*P* < 0.05). Figure S1 shows that the total operational taxonomic units (OTUs) are classified into 16 and 18 phyla obtained from the colonic and caecal digesta, respectively (Figure S1). Among these phyla, Fimicutes was the most abundant phylum, with a relative abundance of 81.87% in the colonic digesta and 80.38% in the cecal digesta. Bacteroidetes was the second most abundant phylum, with an average abundance of 5.61% in the colonic digesta and 5.99% in the cecal digesta. The abundance of Bacteroidetes in the colonic digesta was significantly increased in the HS group (*P* < 0.05) but markedly decreased in the HL group (*P* < 0.05) relative to that in the LC group (Figure S1).

**Table 3:** Effects of high concentrate diet on the average richness and diversity of the bacterial community of the hindgut digesta.

**Items**	**LC**	**HS**	**HL**
**COLONIC DIGESTA**			
ACE	1,332 ± 53	1,222 ± 36	1,131 ± 41^*^
Chao 1	1,398 ± 121	1,208 ± 34	1,117 ± 40^*^
Shannon index	7.47 ± 0.09	7.09 ± 0.13^*^	6.57 ± 0.19^*^
**CAECAL DIGESTA**			
ACE	1,293 ± 37	1,163 ± 48^*^	1,140 ± 64^*^
Chao 1	1,281 ± 35	1,153 ± 47^*^	1,126 ± 64^*^
Shannon index	7.21 ± 0.14	6.83 ± 0.21	6.46 ± 0.34^*^

*ACE, abundance-based coverage estimator*.

* *P < 0.05 vs. LC*.

**Figure 2:**
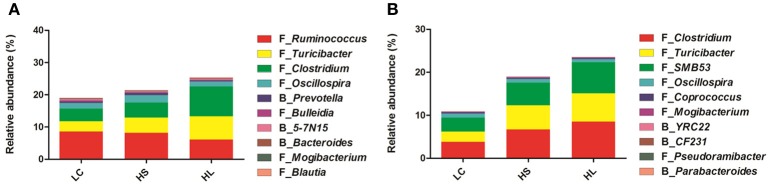
Effect of high-concentrate diet on the changes of microbial taxa (as a percentage of the total sequence). The changes in the percentage of bacterial taxa in colonic **(A)** and caecal **(B)** digesta bacterial community at the genus level (only the taxa whose abundance was significantly affected [*P* < 0.05] by the dietary treatment of top 10 are presented).

The taxa significantly affected by HC diet feeding at the genus level are shown in Figure 2. In the colonic digesta, as the proportion of concentrate increased, the percentages of *Turicibacter, Clostridium, Oscillospira, Prevotella*, and *Bacteroides* also increased. Bycontrast, the abundance of *Ruminococcus, Bulleidia, 5-7N15, Mogibacterium*, and *Blautia* was reduced (Figure 2A). In the cecal digesta, as the proportion of concentrate increased, the percentages of *Clostridium, Turicibacter, SMB53, Mogibacterium, YRC22*, and *Pseudoramibacter* was increased, whereas the proportions of *Oscillospira, Coprococcus, CF231*, and *Parabacteroides* decreased (Figure 2B).

**Figure 3:**
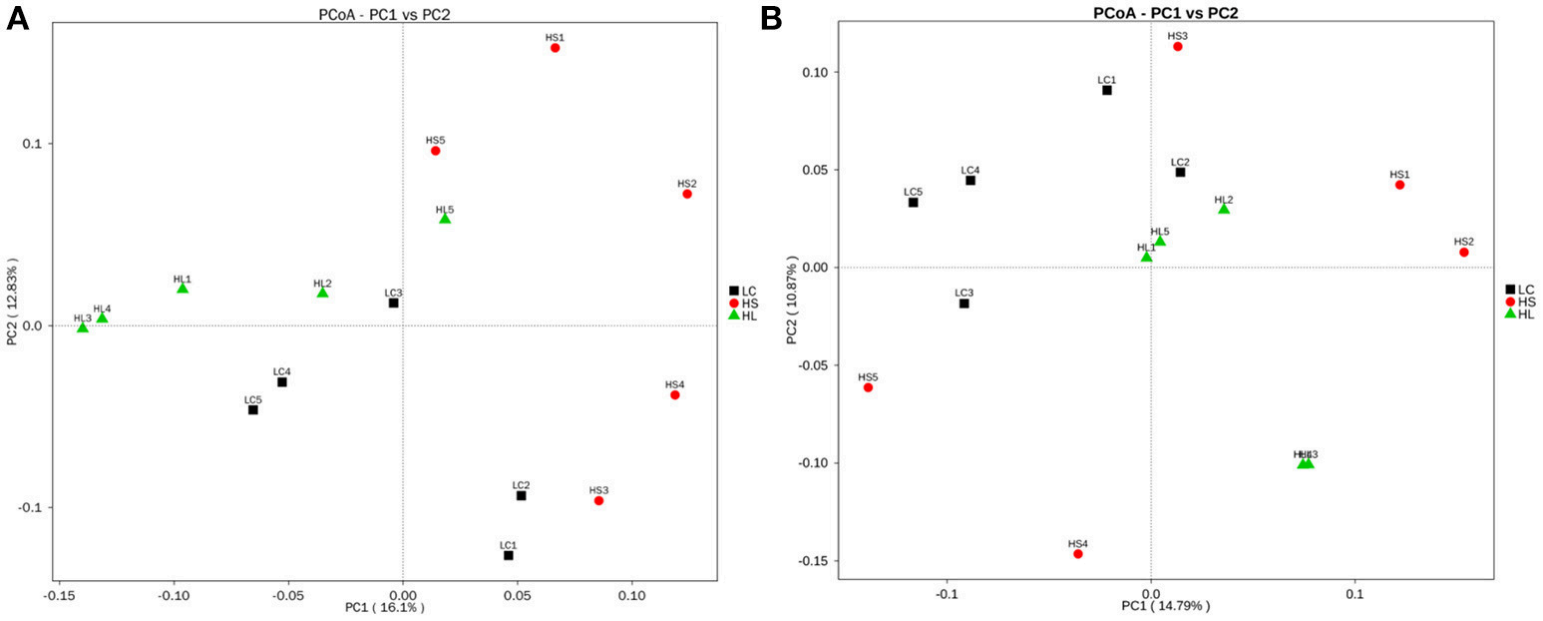
Principal coordinate analysis (PCoA) of the hindgut digesta bacterial communities. **(A)** Unweighted PCoA by colonic digesta bacterial microbiota. **(B)** Unweighted PCoA by caecal digesta bacterial microbiota.

Principal coordinate analysis (PCoA) plots based on unweighted UniFrac distance metrics showed an obvious separation of bacterial communities in colonic digesta among the 3 groups by using the PC1 and PC2 (16.1 and 12.83%, of the explained variance, respectively; Figure 3A). Similarly, the bacterial composition in the cecal digesta of the control group was also clearly separated from the HS and HL groups by PCoA with the PC1 and PC2 (14.79 and 10.87% of the variation, respectively; Figure 3B).

### Correlation between the microbiome and metabolome in the hindgut digesta

**Figure 4:**
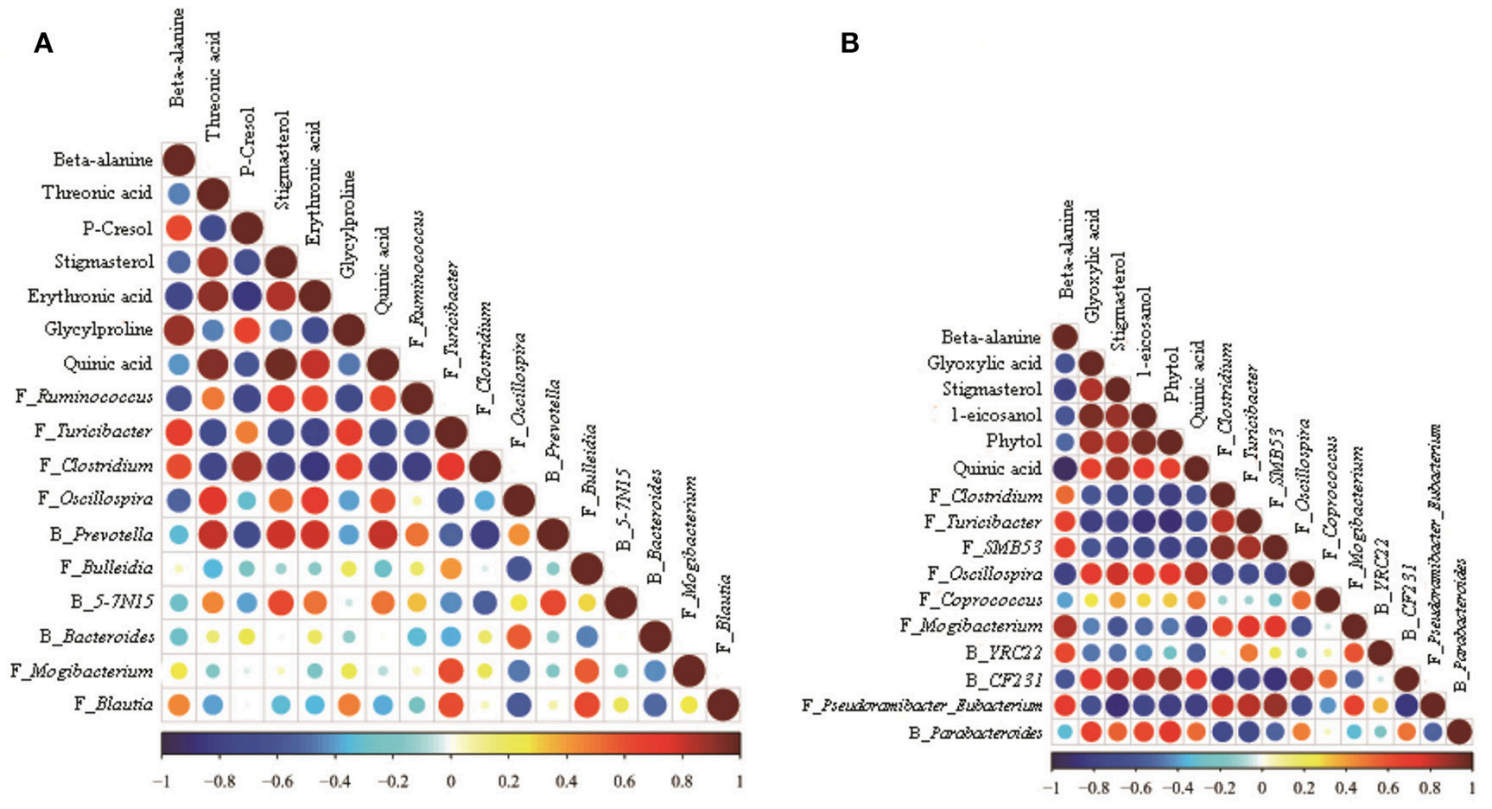
Correlation matrix between the hindgut digesta microbiota (at the genera level; used in Figure 2) affected by the dietary treatment and the potential marker compounds (used in Table 2). Positive correlations are shown in red and negative correlations in blue. Color intensity and the size of the dots are proportional to the correlation values [*r*_*s*_] within a correlation group. **(A)** Colonic digesta, **(B)** Caecal digesta.

The correlations among the microbiome changes and metabolite perturbations in the hindgut digesta were evaluated in this study and are shown in Figure 4. A significant correlation was observed between the perturbed microbiome and the altered metabolite profiles in the hindgut digesta (*r* > 0.60 or < −0.60, *P* < 0.05). Specifically, in the colonic digesta, the correlation analysis indicated the presence of 21 positive or negative correlations between the OTUs and the metabolite (Figure 4A). Meanwhile, in the cecal digesta, 37 positive or negative correlations were determined between the OTUs and the metabolite (Figure 4B).

### Tissue morphology and ultrastructure of the hindgut epithelium

**Figure 5:**
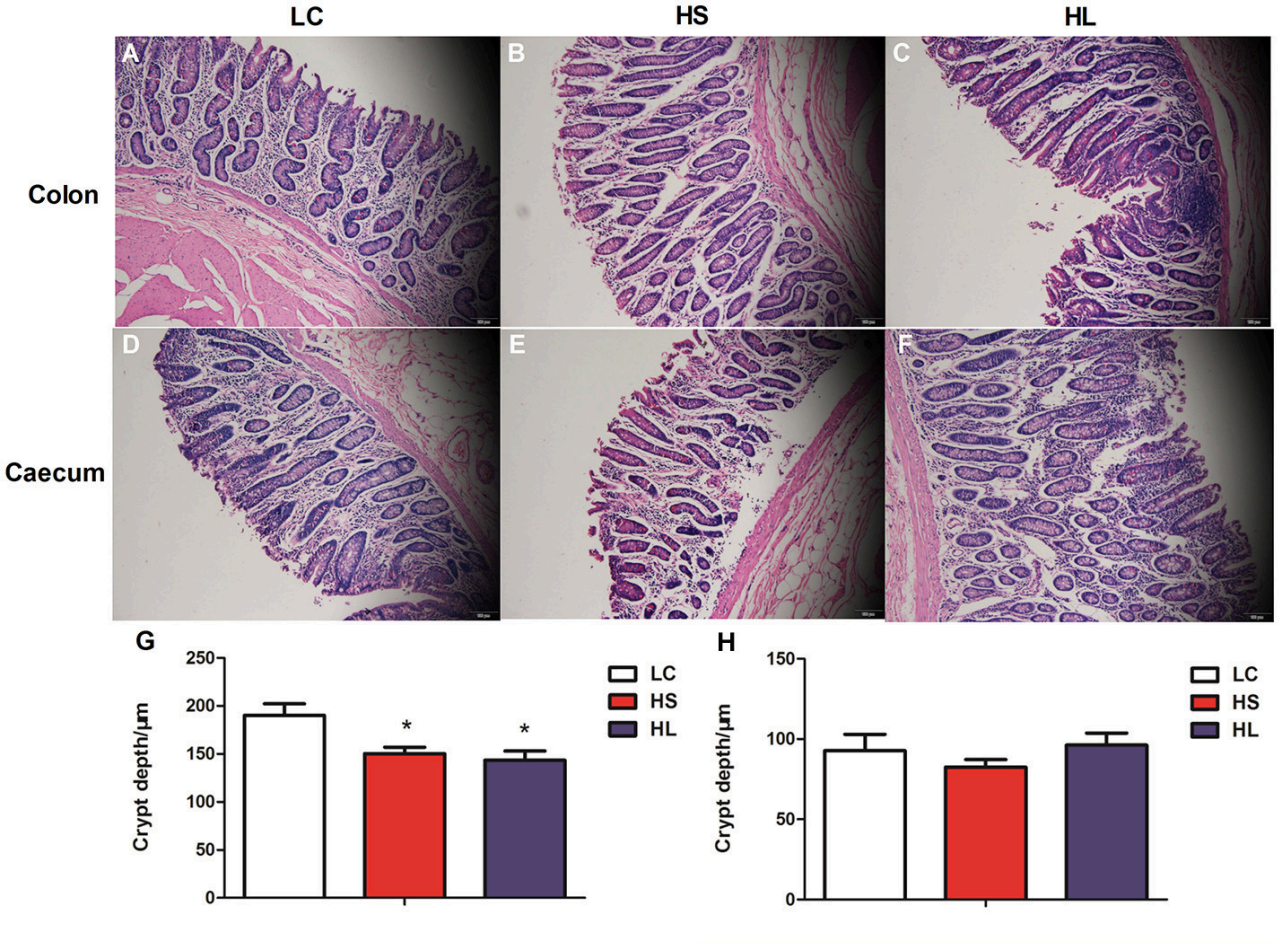
Comparisons of the morphology of the hindgut mucosa between control and dietary treatment goats. Colonic mucosa epithelium from each group was processed for morphological evaluation: colon section of the (**A**, scale bar = 100 μm) LC group; (**B**, scale bar = 100 μm) HS group; (**C**, scale bar = 100 μm) HL group and caecum section of the (**D**, scale bar = 100 μm) LC group; (**E**, scale bar = 100 μm) HS group; (**F**, scale bar = 100 μm). **(G)** Crypt depth of colon. **(H)** Crypt depth of caecum. ^*^ *P* < 0.05 vs. LC.

**Figure 6:**
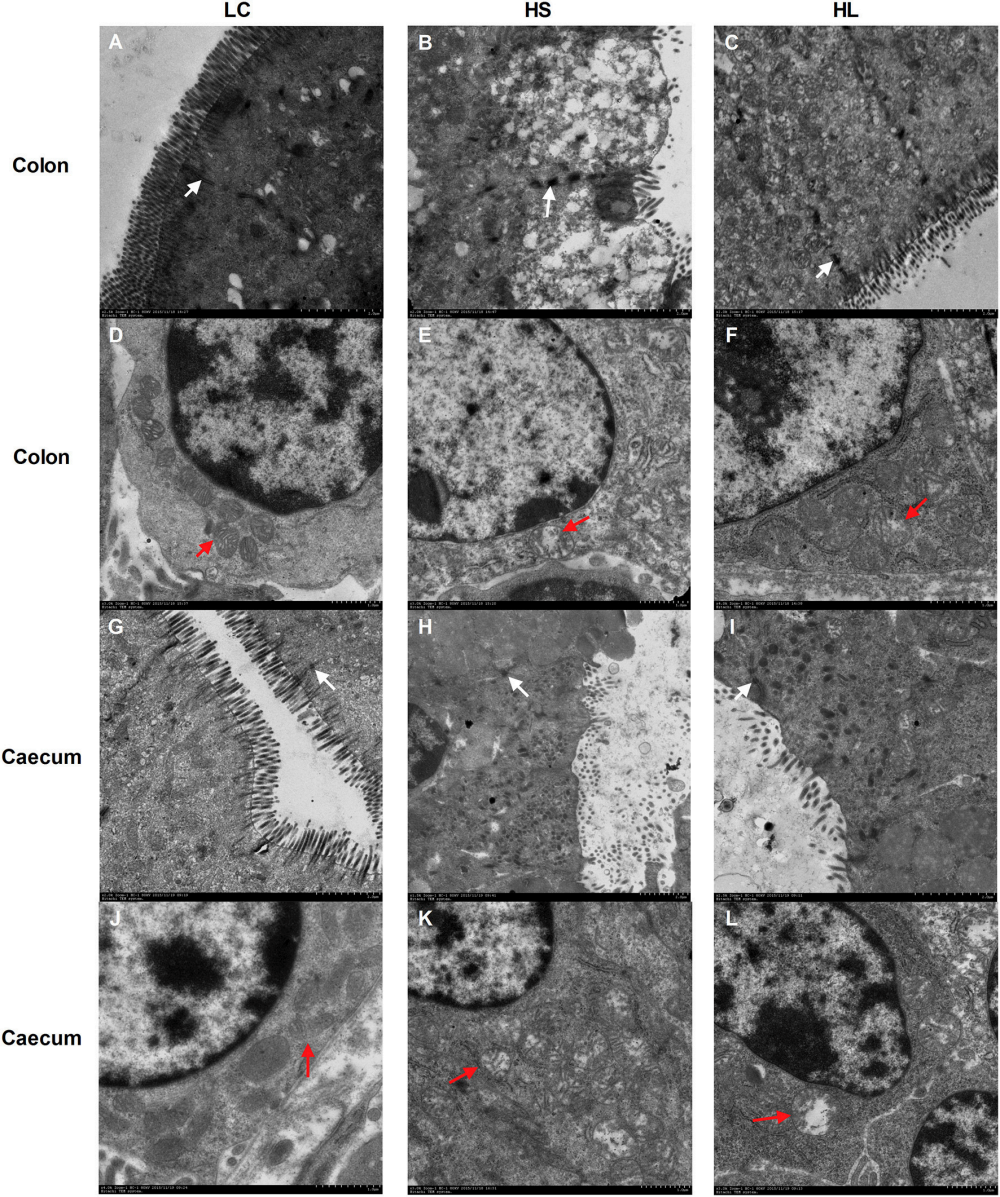
Comparisons of the ultrastructure of the hindgut mucosa between control and diet-treated t goats. Colonic mucosa epithelium from each group was processed for ultrastructure evaluation: colon section of the **(A)** microvilli and TJs of LC group; **(B)** microvilli and TJs of HS group; **(C)** microvilli and TJs of HL group (transmission electron microscopy, × 20,000); **(D)** mitochondria of LC group; **(E)** mitochondria of HS group; **(F)** mitochondria of HL group (transmission electron microscopy, × 10,000); caecum section of the **(G)** microvilli and TJs of LC group; **(H)** microvilli and TJs of HS group; **(I)** microvilli and TJs of HL group (transmission electron microscopy, × 20,000); **(J)** mitochondria of LC group; **(K)** mitochondria of HS group; **(L)** mitochondria of HL group (transmission electron microscopy, × 10000). White arrow indicates the location of the TJs and red arrow indicates the mitochondria.

Representative light micrographs of the cross-section of the hindgut epithelium are presented in Figure 5. The results revealed that the integrity of the epithelial cell morphology of the colonic and cecal epithelial structures was preserved in the LC goats (Figures 5A,D). Meanwhile, both the HS and HL goats exhibited desquamation in the colonic and cecal epithelial mucosa (Figures 5B,C,E,F). The colonic crypt depth of the HS and HL goats was significantly (*P* < 0.05) lower than that of the LC control goats (Figure 5G), whereas no significant difference (*P* < 0.05) was found in the crypt depth of the cecal epithelium among the 3 groups (Figure 5H). The ultrastructure of the hindgut epithelium is presented in Figure 6. The microvillus clusters in the LC goats were clear and well-organized (Figures 6A,G), whereas the microvilli in the hindgut mucosa of the HS and HL goats were sloughy and irregularly distributed (Figures 6B,C,H,I). The hindgut epithelium of the HS and HL goats showed damaged intercellular tight junctions and wider intercellular space (Figures 6B,C,H,I), whereas those of the LC goats exhibited integrity and a normal tight junction structure (Figures 6A,G). The LC goats also exhibited normal mitochondrial structure (Figures 6D,J), whereas the HS- and HL-fed goats showed apparent mitochondrial swelling (Figures 6E,F,K,L).

### Gene expression in the hindgut mucosa

**Figure 7:**
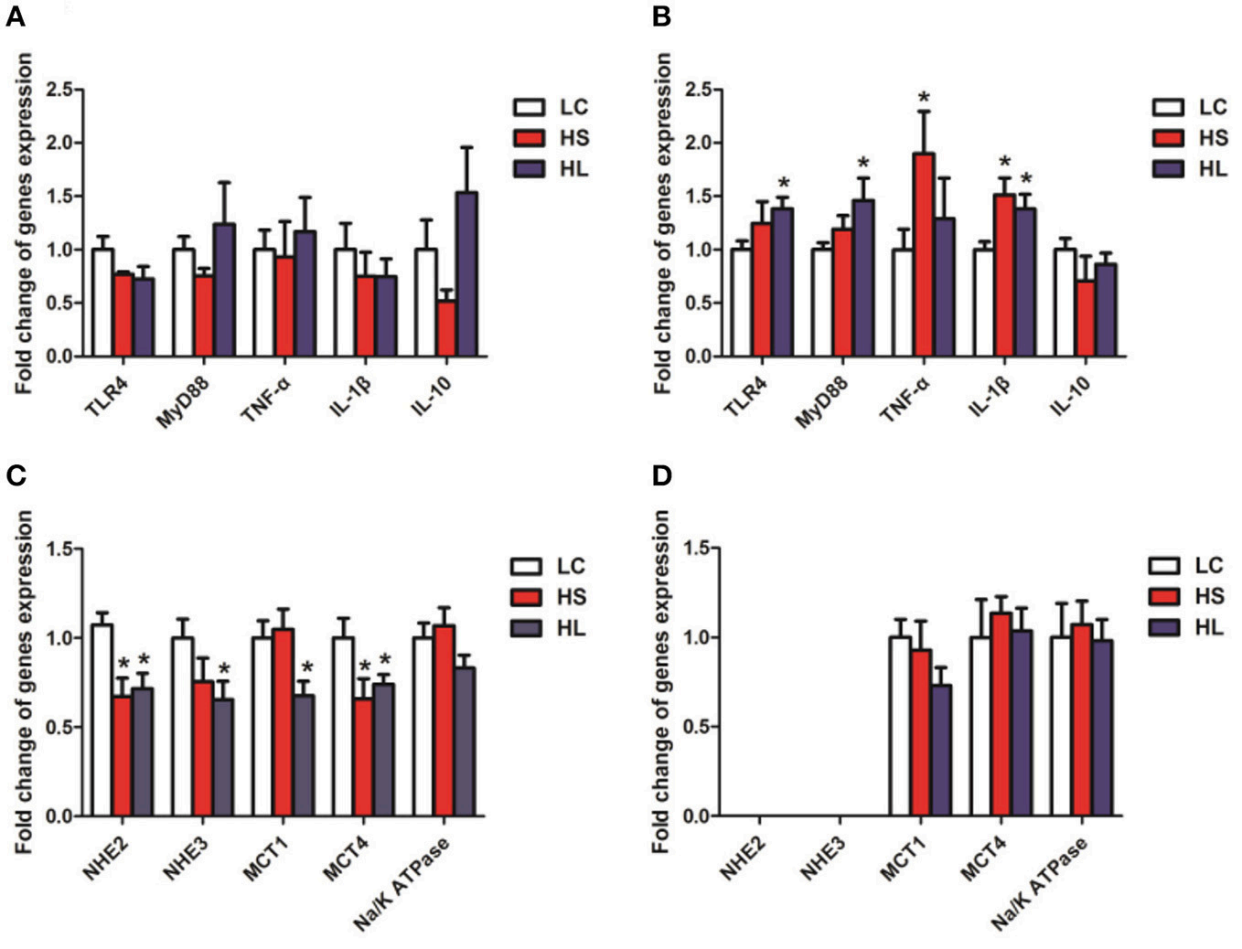
Gene expression and oxidative stress parameters in the hindgut mucosa. β-actin was used as the reference gene for gene expression. **(A,C)** Gene expression in the colonic mucosal; **(B,D)** Gene expression in the caecal mucosal. ^*^ *P* < 0.05 vs. LC.

The relative mRNA expression levels of the genes functionally associated with inflammation and nutrients transport were evaluated by real-time quantitative polymerase chain reaction (RT–qPCR) analysis. The expression levels of the genes related to the inflammatory response, including *TLR4, MYD88, TNF-*α, *IL-1*β, *and IL-10* were not significantly altered in the colonic mucosa by the HC diet (Figure 7A). By contrast, most genes were markedly upregulated in the cecal mucosa by the HC diet (Figure 7B). Compared with the control group, the HL group showed upregulated expression of *TNF-*α and *IL-1*β in the cecal mucosa (*P* < 0.05); meanwhile, the HL group showed upregulated expression of *TLR4, MYD88*, and *IL-1*β in (*P* < 0.05). In addition, the expression levels of *NHE2* and *MCT4* were downregulated in the colonic mucosa of the HS goats (*P* < 0.05). Compared with the LC goats, the HL goats had lower *NHE2, NHE3, MCT1*, and *MCT4* expression levels in the colonic mucosa (*P* < 0.05). No significant difference (*P* > 0.05) in the expression levels of *MCT1* and *MCT4* in the cecal mucosa was determined among the 3 groups (Figure 7D). *Na*^+^*/K*^+^ *ATPase* mRNA expression was not altered in the colonic and cecal mucosa by the HC diet (Figures 7C,D). Moreover, *NHE2* and *NHE3* expression in the cecal mucosa was too low to be detected (Figure 7D).

## Discussion

### Alterations in hindgut fermentation and microbial composition during HC diet feeding

Over the recent decades, milk production per dairy cattle has markedly increased. This increase may be attributed to feeding rations with a greater proportion of grains and less forage (Ellis et al., 2012; Metzler-Zebeli et al., 2013a). Digestion of a high-grain diet leads to deficient buffering capacity in the rumen, boosts the accumulation of organic acids, and ultimately increases the incidence of acidosis (Owens et al., 1998). The increased amount of fermentable carbohydrates passes through the forestomach to the hindgut and then accelerates hindgut fermentation (Li et al., 2012). The current study confirmed that feeding an HC diet to lactating dairy goats significantly increases total SCFA and free LPS concentration in the lumen of the hindgut, indicating the impairment of fermentation and dysbiosis in the hindgut of goats.

The alterations in the bacterial community in ruminal and hindgut digesta and their adaptation to different dietary regimens have been widely reported in ruminants that were fed an HC diet for a short period (Metzler-Zebeli et al., 2013b; Liu et al., 2014, 2015; Wetzels et al., 2015). However, the shift of the bacterial community in the hindgut digesta of the goats in response to HC dietary changes for a long period remains undetermined. This study indicated that compared with the LC control goats, both the HS and HL goats exhibit reduced bacterial richness and diversity, as indicated by the ACE, Chao 1, and Shannon indices. This finding suggests that the HC diet exerts a considerably negative effect on the biodiversity of the hindgut ecosystem of lactating goats. The results of the unweighted UniFrac PCoA and AMOVA further revealed the difference in bacterial community composition between the control and the HC groups and showed that feeding an HC diet altered the composition of the bacterial community in the hindgut digesta. The alterations of the microbial population structure and diversity are associated with the accumulation of fermentable substrates as well as some toxic products in ruminants fed with a high-grain diet (Zened et al., 2013).

In the present study, univariate statistical analysis indicated that the HC diet supports a large population of various bacterial taxa, including *Turicibacter, Clostridium, Oscillospira, Prevotella*, and *Bacteroides* in colonic digesta. *Turicibacter* is a relatively unknown genus; regardless, recent reports suggest the presence of *Turicibacter* bacteria in the rumen and feces of cattle (Shanks et al., 2011; Mao et al., 2013). The increase in the percentage of *Prevotella* in the colonic digesta of the goats fed with an HC diet was consistent with the increase in the starch content of the hindgut digesta of the goats fed with an HC diet. We also found that Gram-negative *Bacteroides* were stimulated by the HC diet. A previous study inferred that a majority of LPS produced in the digesta derived from *Bacteroides* spp. (Khafipour et al., 2009). In the current study, the increase in the *Bacteroides* population was accompanied by a significant increase in free LPS content in the colonic digesta. In the cecal digesta, the HC diet increased the abundance of the *Clostridium, Turicibacter, SMB53, Mogibacterium, YRC22*, and *Pseudoramibacter* taxa. Consistent with the report by Liu et al. (2014), our study findings indicated that high-grain diets increased the relative abundance of *Mogibacterium* in the cecum of goats. The role of the *YRC22* species in ruminants remains undetermined (McCann et al., 2016), however, a previous study demonstrated that it is increased in the rumen of cows with SARA.

In the current study, we also found that additional bacteria, including those from the *Ruminococcus, Bulleidia, 5-7N15, Mogibacterium*, and *Blautia* taxa were significantly reduced in the colonic digesta of the HC-fed goats compared with the controls. *Ruminococcus* is the most dominant genus found in the large intestine of healthy sheep (Wang et al., 2016) and plays an important role in degrading starch (Flint et al., 2008; Chassard et al., 2012). *Mogibacterium* is associated with ammonia assimilation (Nakazawa et al., 2000), thus, the reduced abundance of *Mogibacterium* in goats fed with an HC diet can potentially contribute to the accumulation of ammonia in the digestive tracts and then pose potential threats to gut health. A meta-analysis of 5 microbiota studies in patients with inflammatory bowel disease demonstrated that the population of *Oscillospira* is significantly reduced in patients with Crohn's disease (Walters et al., 2014). In the present study, HC-fed goats exhibited an inflammatory response, as indicated by the expression of inflammation-associated genes and the alterations in the histological structure of the cecal mucosa. This finding also agrees with a previous study (Tao et al., 2014a). In addition, *Parabacteroides* reportedly prevent mice from being susceptible to the development of metabolic syndrome (Economopoulos et al., 2016). By contrast, the decrease in *Parabacteroides* in HC-fed animals may be involved in the development of fermentation disorders in the rumen as well as in the hindgut.

### Shift of metabolomics and correlation analysis during HC feeding

Accumulating evidence suggests that metabolic changes associated with intestinal microbiome perturbations are important risk factors affecting the generation of a harmful intestinal microenvironment (Levy et al., 2015). In the current study, PLS-DA analysis identified 7 compounds in colonic digesta as the most important factors for distinguishing the control and HC-fed goats: beta-alanine, threonic acid, p-cresol, stigmasterol, erythronic acid, glycylproline, and quinic acid. Beta-alanine, not a precursor for the biosynthesis of major proteins or enzymes, is normally metabolized into acetic acid. The concentration of beta-alanine was increased by the HC diet, which was consistent with the increase in the acetate level. Studies identified p-cresol as a toxin in the sheep urine (White et al., 1950) and as a metabolic troublemaker and a genotoxic agent toward colonocyte (Andriamihaja et al., 2015). Moreover, the production of p-cresol was shown to be associated with *Lactobacillus* sp. (Yokoyama and Carlson, 1981). However, in the current study, the HC diet exerted no effect on the proportion of *Lactobacillus* in general, suggesting that bacteria species other than *Lactobacilli* were involved in p-cresol production. Indeed, our results showed that the p-cresol level was significantly correlated with the abundance of *Ruminococcus* (negative correlation), *Clostridium* (positive correlation), and *Prevotella* (negative correlation). Glycylproline, a stable substrate for the di/tri/oligopeptide transport system, was used as an affinity probe (Moore et al., 2000). Glycylproline significantly reduced the permeability of cefadroxil, a bactericidal antibiotic (Posada and Smith, 2013). Correlation analysis indicated a significant correlation between the level of glycylproline and the density of *Turicibacter* (positive correlation) and *Clostridium* (positive correlation). Among the compounds affected by the HC diet, threonic acid and erythronic acid are closely related to vitamin C, a common antioxidants (Eberhardt et al., 2000). Threonic acid is a metabolite of vitamin C, whereas erythronic acid is a stereoisomer of vitamin C. Threonic acid and erythronic acid levels were significantly lower in the HC-fed group than in the control group and were correlated with the abundance of *Turicibacter* (negative correlation) and *Clostridium* (negative correlation). Our study revealed that the levels of quinic acid and stigmasterol were significantly decreased. These decreases were markedly correlated with the abundance of *Turicibacter* (negative correlation) and *Clostridium* (negative correlation).

Six compounds were differentially expressed in cecal digesta by the HC diet, namely, beta-alanine, glyoxylic acid, stigmasterol, 1-eicosanol, phytol, and quinic acid. Among these compounds, stigmasterol and quinic acid were also differentially expressed in the colonic digesta, indicating the common metabolic pathways altered by the HC diet in the colon and cecum. In cecal digesta, the level of stigmasterol was positive correlated with the abundance of the *Oscillospira* and *CF231*. Moreover, the level of stigmasterol was negative correlated with the following bacterial genera: *Clostridium, Turicibacter, SMB53*, and *Pseudoramibacter*. However, the level of quinic acid was positive correlated with the abundance of *Oscillospira and CF231*. In addition, the level of quinic acid was negative correlated with the abundance of *Turicibacter, SMB53, Mogibacterium*, and *Pseudoramibacter*. Excessive pantothenic acid induces the accumulation of fatty acid, which eventually leads to metabolic syndrome (Tahiliani and Beinlich, 1991). In the current study, the HC diet increased beta-alanine but decreased glyoxylic acid in the cecal digesta, indicating that the conversion efficiency from volatile fatty acids to carbohydrates was decreased and resulted in metabolic disorder in the ruminants. Our finding regarding phytol is consistent with that of a previous study (Silva et al., 2014), which found that phytol attenuates inflammatory response by partly inhibiting neutrophil migration by decreasing the levels of in *IL-1*β and *TNF-*α. Our finding indicates that phytol is reduced and the relative expression levels of *IL-1*β and *TNF-*α are considerably increased by the HC diet. These results imply that the HC diet could induce inflammation in the cecal mucosa.

## Conclusion

This study revealed that feeding an HC diet to lactating goats alters fermentation pattern, bacterial dysbiosis, and metabolic perturbations. The negative effects of feeding an HC diet on the biodiverse ecosystem as well as the increased abnormal products in the hindgut threaten the health and physiological functions of the gut. Consistently, we also found significant changes in gene expression involved in inflammatory response and nutrient transport in the hindgut epithelium of the HC-fed animals. In general, this study provides a comprehensive picture of the biochemical and microbial function in the hindgut of ruminants fed with an HC diet and elucidates the etiology of metabolic disorders occurring in the hindgut. This study also provides guidelines to sustain the balance between high production and animal welfare.

## Materials and methods

### Ethics

All animal procedures were approved by the Institutional Animal Care and Use Committee of Nanjing Agricultural University. The protocol of this study was reviewed and specifically approved with the project number 2011CB100802. The slaughter and sampling procedures strictly followed the “Guidelines on Ethical Treatment of Experimental Animals” (2006) no. 398 set by the Ministry of Science and Technology, China and the “Regulation regarding the Management and Treatment of Experimental Animals” (2008) no. 45 set by the Jiangsu Provincial People's Government.

### Animals and experimental procedures

Fifteen mid-lactating goats of ~46.3 ± 1.4 kg body weight were used in this study. Two weeks before the start of this experiment, goats were offered free access to a diet containing a forage-to-concentrate ratio (F: C) of 65:35 to ensure adaptation to the diet. After dietary adaptation, goats were randomly allocated to two groups. One group was fed a control diet composed of 65% forage and 35% mixed concentrate (low concentrate group, LC), while the other group received a high-grain diet containing 65% mixed concentrate and 35% forage (high concentrate, long-term group, HL) for 19 weeks. After a 13-week feeding period, we randomly chose 5 goats from the LC group and assigned them to a new group fed the high-grain diet containing 65% mixed concentrate and 35% forage (high concentrate, short-term group, HS), for 6 weeks, including 2 weeks of dietary adaptation. The details of the diet components and nutrient compositions are given in Table S1. The animals had free access to water during the experimental period.

### Sample collection

At the end of the experiment, after an overnight fast, all goats were killed by I.V. injections of xylazine [0.5 mg (kg body weight)^−1^; Xylosol; Ogris Pharme, Wels, Austria] and pentobarbital [50 mg (kg body weight)^−1^; Release; WDT, Garbsen, Germany]. Immediately after death, the hindgut (colon and caecum) mucosal tissues were carefully harvested. Digesta from the proximal colon and caecum was aseptically collected and kept on ice until being stored at −20°C. Within 20 min of death, a segment of the colonic and caecal mucosa from the same position in each animal was collected. The colonic and caecal epithelium was separated from the muscular layers by blunt dissection and immediately washed three times in ice-cold PBS. The tissue samples were frozen immediately in liquid nitrogen and then used for further analysis.

### Physiological parameters measurements

The SCFA of the hindgut digesta was analyzed by gas chromatography (GC-14B; Shimadzu, Kyoto, Japan; capillary column: 30 m × 0.32 mm × 0.25 μm; temperature of the column 110°C, temperature of the injector 180°C, temperature of the detector 180°C). Lactate levels in the hindgut digesta were determined using a Lactate Assay Kit (Nanjing Jiancheng Bioengineering Institute, Nanjing, China). Free LPS content in the hindgut was measured via a Chromogenic End-point Tachypleus Amebocyte Lysate Assay Kit (Xiamen Horseshoe Crab Reagent Manufactory, Ximen, China) with a minimum detection limit of 0.1 EU/mL. Pretreated supernatants were diluted until their LPS concentrations were in the range of 0.1 to 1 EU/mL relative to the reference endotoxin.

### Histological measurements

Specimens from the intestinal wall of the colonic and caecal mucosa were prepared for histological examination by fixing in 4% formaldehyde-buffered solution, embedding in paraffin, and sectioning. Specimens were examined for injury after hematoxylin and eosin (H&E) staining as previously described (Yue et al., 2012). The depth of total crypt was measured using the Mirax Viewer Software (Version 1.12.22.0, Carl Zeiss, Göttingen, Germany) from 20 crypts per animal. Colonic and caecal mucosa tissue samples were separated and fixed immediately with 2.5% glutaraldehyde, post-fixed with 1% osmium tetroxide, and embedded in resin. Ultrathin sections were cut and stained with uranyl acetate and lead citrate. Epithelial tissues ultrastructure was examined with a transmission electron microscope (Hitachi H-7650, Hitachi Technologies, Tokyo, Japan).

### RNA isolation, cDNA synthesis and real-time PCR

Hindgut mucosal tissue was quickly collected, immediately frozen in liquid nitrogen and stored at −80°C until RNA isolation. Total RNA was extracted from colonic and caecal samples with the TRIzol reagent (15596026; Invitrogen, Shanghai, China). The concentration and quality of the RNA were measured with a NanoDrop ND-1000 Spectrophotometer (Thermo Fisher Scientific, Walthan, MA, USA). Next, 2 μg of total RNA was treated with RNase-Free DNase (M6101; Promega, Madison, WI, USA) and reverse transcribed according to manufacturer's instructions. Two microliters of diluted cDNA (1:20, v/v) was used for real-time PCR, which was performed in an Mx3000P (Stratagene, La Jolla, CA, USA). β-actin, whose expression is not affected by the experimental factors, was chosen as the reference gene. All the primers chosen to study the expression of genes related to inflammation and volatile fatty acids transport are listed in Table S2 and were synthesized by Generay Company (Shanghai, China). The method of 2^−ΔΔ*Ct*^ was used to analyze the real-time PCR data, and gene mRNA levels were expressed as the fold change relative to the mean value of the control group.

### Mucosal microbial DNA extraction 16S rDNA gene amplicon pyrosequencing

A total of 0.25 g of wet colonic and caecal digesta was used for DNA extraction. The DNA was extracted by a bead-beating method using a QIAamp DNA Stool Mini Kit (Qiagen, Hilden, Germany) according to the manufacturer's instructions. Briefly, colonic and caecal digesta samples were disrupted in an ASL buffer and homogenized with 100 mg of zirconium beads (0.1 mm) in a Mini-Beadbeater-1 (Biospec Products Inc. Bartlesville, OK, USA) at a rate of 4,800 rpm/min four times for 30 s each time at room temperature. Lysozyme was then added at a final concentration of 20 mg/mL (Sigma), and the suspension was incubated at 37°C for 40 min to improve lysis efficiency. Subsequently, the mixture was incubated in a 95°C water bath for 5 min to further increase the amount of total DNA extraction. PCR amplification, sequencing and sequencing data processing were performed as described in our previous study (Hua et al., 2017).

### Metabolite profiling of the hindgut digesta

A 40 mg of intestinal contents sample and 400 μL of 80% methanol were successively added to a tube. The mixture was sonicated at 4°C, and placed at −20°C for 2 h prior to centrifugation at 16,000 g and 4°C for 15 min. Next, 320 μL of supernatant was transferred into a new tube. The procedure was repeated by adding 320 μL of methanol. Another 320 μL of supernatant was transferred and combined with the first extraction. Then, 250 μL of the mixture was added to a GC vial, containing 10 μL of internal standards (0.05 mg/mL ^13^C3-^15^N-L-alanine, ^13^C5-^15^N-L-valine, ^13^C6-^15^N-L-leucine, ^13^C6-^15^N-L-isoleucine). The mixture was dried under a gentle nitrogen stream. The GC vial with dry residue was added to 30 μL of 20 mg/mL methoxyamine hydrochloride in pyridine. The resultant mixture was vortex-mixed vigorously for 30 s and incubated at 37°C for 90 min. A 30 μL of BSTFA (with 1% TMCS) was added into the mixture, which was derivatized at 70°C for 60 min. Metabolomics instrumental analysis was performed on an Agilent 7890A (Agilent Technologies Inc., Santa Clara, CA, USA) gas chromatography system coupled to an Agilent 5975C inert MSD system. A HP-5ms fused-silica capillary column (30 m × 0.25 mm × 0.25 μm; Agilent J&W Scientific, Folsom, CA) was used to separate the derivatives. Helium (>99.999%) was used as a carrier gas at a constant flow rate of 1 mL/min through the column. Injection volume was 1 μL in splitless mode, and the solvent delay time was 6 min. The initial oven temperature was held at 70°C for 2 min, ramped to 160°C at a rate of 6°C/min, to 240°C at a rate of 10°C/min, to 300°C at a rate of 20°C/min, and finally held at 300°C for 6 min. The temperatures of injector, transfer line, and electron impact ion source were set to 250, 290, and 230°C, respectively. The impact energy was 70 eV, and data were collected in a full scan mode (m/z 50–600). The differential metabolites were determined by the combination of the Variable importance in the projection (VIP) value (>1.4) of OPLS-DA model and the *p-*values (<0.05) from two-tailed Student's *t*-test on the normalized peak intensities. Fold change was calculated as the binary logarithm of the average normalized peak intensity ratio between Group 1 and Group 2, where the positive value means that the average mass response of Group 1 is higher than Group 2. The structural identification of the differential metabolites was performed as follows. The AMDIS software was applied to deconvolute the mass spectra from the raw GC-MS data, and the purified mass spectra were automatically matched with an in-house standard library including retention time and mass spectra, Golm Metabolome Database, and Agilent Fiehn GC/MS Metabolomics RTL Library. Partial least squares discriminant analysis (PLS-DA) was conducted on metabolite data. PLS-DA is a frequently used PLS-based classification method where the response variable is a categorical one (dummy variables describing the categories) expressing the class membership of the statistical units.

### Data analysis

The statistical analyses were carried out with tests using the SPSS software package (SPSS v. 16, SPSS Inc., Chicago, IL, USA). The goat was the experimental unit for all comparisons, and diet was regarded as the fixed effect. The normality of the distribution of variables was tested by the Shapiro–Wilk test. The independent samples *T*-test procedure was used to analyze the variables found to have a normal distribution. The variables found to have a non-normal distribution were analyzed using the Kruskal–Wallis test procedure. Significance was declared at *P* < 0.05.

The peak information (named matrix X) was imported to Simca-P (version 11.0, Umetrics AB, Umeå, Sweden), where a multivariate statistical analysis, such as PLS-DA, was performed. All data were mean-centered and unit variance (UV)-scaled prior to multivariate statistical analysis. The quality of the models is described by the R^2^X or $R^2^Y and Q^2^ values. R^2^X or R^2^Y is defined as the proportion of variance in the data explained by the models and indicates the goodness of fit. Q^2^ is defined as the proportion of variance in the data predictable by the model and indicates the predictability of current model, calculated by a cross-validation procedure. In order to avoid model over-fitting, a default 7-round cross-validation in Simca-P was performed throughout to determine the optimal number of the principal components. The values of R^2^X, R^2^Y, and Q^2^ were used as indicators to assess the robustness of a pattern recognition model.

Double dendograms were constructed using the comparative functions and multivariate hierarchical clustering methods of Hemi (Deng et al., 2014), on the basis of the abundances of the bacterial groups at different taxonomic levels. The correlation was made by the corrplot package of the R software. A value of *P* < 0.05 was regarded as statistically significant.

## Author contributions

ST performed the experiment and drafted the manuscript; PT, YL, JT, CH, and YG performed the experiment and analyzed the data; RC and RZ contributed to experimental design and manuscript revision; YN conceived the idea, designed the experiment and finalized the manuscript. All authors have approved the final version of the manuscript and agree to be accountable for all aspects of the work. All persons designated as authors qualify for authorship, and all those who qualify for authorship are listed.

### Conflict of interest statement

The authors declare that the research was conducted in the absence of any commercial or financial relationships that could be construed as a potential conflict of interest.

## Supplementary material

Figure S1 Effect of the high-concentrate diet on changes of phyla (as a percentage of the total sequence). The changes in the percentage of bacterial taxa in colonic (A) and caecal (B) digesta bacterial community at the phyla level [only the taxa whose abundance was significantly affected (*P* < 0.05) by the dietary treatment are presented].

## Abbreviations

LC: low concentrate
HC: high-concentrate
HS: high concentrate, short-term
HL: high concentrate, short-term
LPS: lipopolysaccharide
VFA: volatile fatty acid
TJ: tight junction
SARA: subacute ruminal acidosis
SCFA: short-chain fatty acid
VIP: variable importance in the projection
ACE: abundance-based coverage estimator
OTU: operational taxonomic unit
RT-qPCR: real-time quantitative polymerase chain reaction
SOD: superoxide dismutase
GPX: glutathione peroxidase
MDA: malondialdehyde
T-AOC: total antioxidant capacity
TLR4: toll-like receptor 4
MYD88: myeloid differentiating factor 88
TNF-α: tumor necrosis factor alpha
IL-1β: interleukin-1 beta
IL-10: interleukin-10
NHE: Na^+^/H^+^ hydrogen exchanger
MCT: monocarboxylate transporter.
