# Supplementary figures and images for: Microbiome-Metabolome Responses to a High-Grain Diet Associated with the Hind-Gut Health of Goats
Source: Front Microbiol. 2017 Sep 14;8:1764. doi: 10.3389/fmicb.2017.01764 (PMC5603706; doi:10.3389/fmicb.2017.01764)

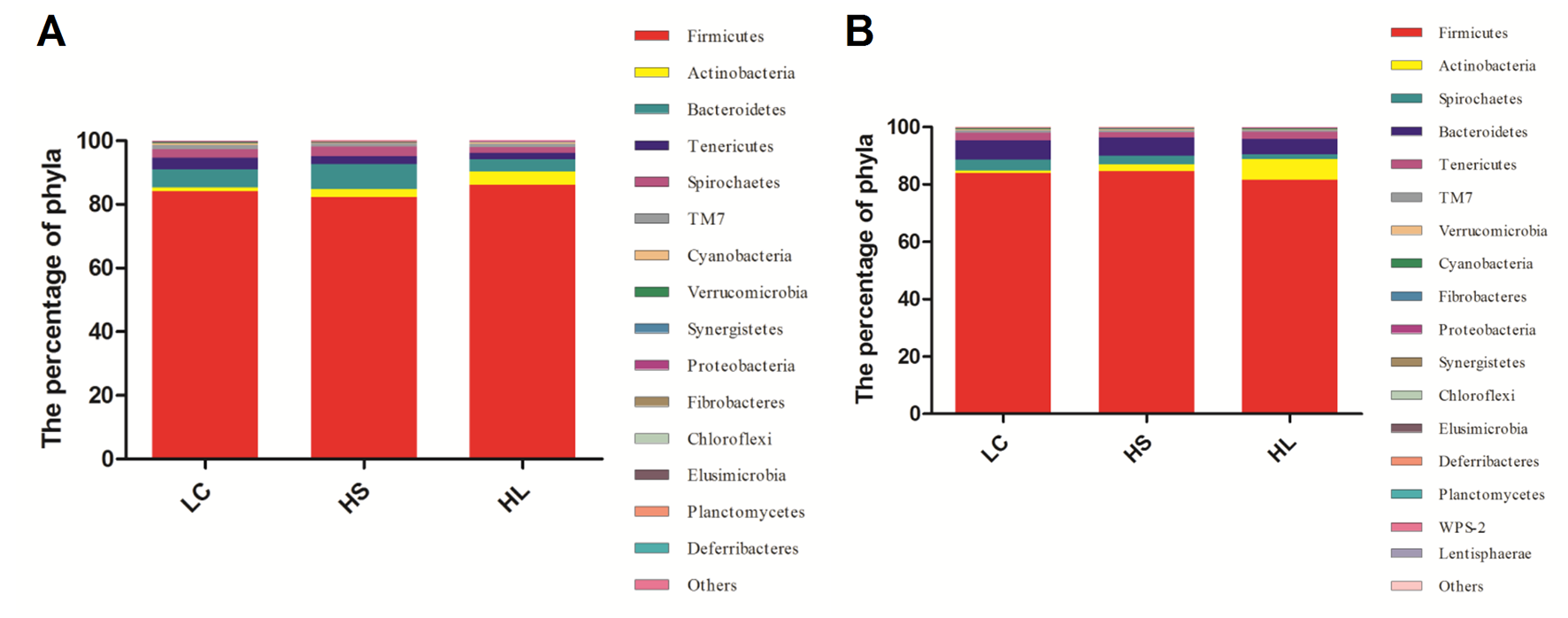

Supplement: Figure S1 — Effect of the high-concentrate diet on changes of phyla (as a percentage of the total sequence). The changes in the percentage of bacterial taxa in colonic (A) and caecal (B) digesta bacterial community at the phyla level [only the taxa whose abundance was significantly affected (P < 0.05) by the dietary treatment are presented]. [file Image2.TIF]
