# Supplementary material for: Microbiome-Metabolome Responses to a High-Grain Diet Associated with the Hind-Gut Health of Goats
Source: Front Microbiol. 2017 Sep 14;8:1764. doi: 10.3389/fmicb.2017.01764 (PMC5603706; doi:10.3389/fmicb.2017.01764)
Supplement: Supplementary file 3 [file Table2.doc]

**Table S2 PCR primer sequences of the target genes**

| Target gene | Genbank accession | PCR products (bp) | Primer sequences |
| --- | --- | --- | --- |
| β-actin | AF_481159 | 260 | F: 5'-CGGGATCCATCCTGCGTCTGGACCTG -3' |
|  |  |  | R: 5'-GGAATTCGGAAGGAAGGCTGGAAGAG -3' |
| TLR4 | JQ342090.1 | 195 | F: 5'-GTTTCCACAAGAGCCGTAA-3' |
|  |  |  | R: 5'-TGTTCAGAAGGCGATAGAGT-3' |
| MyD88 | JQ308783.1 | 98 | F: 5'-ACAAGCCAATGAAGAAAGAG-3' |
|  |  |  | R: 5'-GAGGCGAGTCCAGAACC-3' |
| TNF-α | AF276985.1 | 173 | F: 5'-CAAGTAACAAGCCGGTAGCCC-3' |
|  |  |  | R: 5'-CCTGAAGAGGACCTGCGAGTAG-3' |
| IL-1β | D63351.1 | 172 | F: 5'-GAAGAGCTGCACCCAACA-3' |
|  |  |  | R: 5'-CAGGTCATCATCACGGAAG-3' |
| IL-10 | DQ837159.1 | 239 | F: 5'-TTAAGGGTTACCTGGGTTGC-3' |
|  |  |  | R: 5'-CCCTCTCTTGGAGCATATTGA-3' |
| NHE2 | XM_604493.9 | 93 | F: 5'-GGTCATATTCTTCACTGTCTTC-3' |
|  |  |  | R: 5'-GCTTGCTGCTTCTTATTGG-3' |
| NHE3 | NM_001192154.1 | 108 | F: 5'-AAGAACCTGTTTGTCAGCACCAC-3' |
|  |  |  | R: 5'-TTCACTTCTCTTCACCTTCAGCC-3' |
| MCT1 | AB231662.1 | 168 | F: 5'-CTTGGCAG ACCTTTATCCTC-3' |
|  |  |  | R: 5'-CTCCACAATGGTCACCAATCC-3' |
| MCT4 | NM_001109980.1 | 118 | F: 5'-CTACAGAGCCTGAGAAGAACGG-3' |
|  |  |  | R: 5'-GCTAAATACGAGCGTTGACGG-3' |
| Na^+^/K^+^ ATPase | NM_001076798.1 | 137 | F: 5'-CCTCGAAATCCATTGCTTATACC-3' |
|  |  |  | R: 5'-GACCATGTCCGTTCCCAAGT-3' |

*TLR 4*: Toll-like receptor 4; M*YD88*: Myeloid differentiating factor 88; *TNF-α*: Tumor necrosis factor alpha; *IL-1β*: Interleukin-1 beta; *IL-10*; Interleukin-10; *NHE*: Na^+^/H^+^ hydrogen exchanger; *MCT*: monocarboxylate transporter.
